# Variations in the MHC Region Confer Risk to Esophageal Squamous Cell Carcinoma on the Subjects from High-Incidence Area in Northern China

**DOI:** 10.1371/journal.pone.0090438

**Published:** 2014-03-04

**Authors:** Fang-Fang Shen, Wen-Bin Yue, Fu-You Zhou, Ying Pan, Xue-Ke Zhao, Yan Jin, Xin Song, Bei Li, Xue-Na Han, Sa Tang, Yan Li, Guo Yuan, Li-Sha Chen, Ya-Li Liu, Yan-Long Hu, Xiu-Min Li, Jing-Li Ren, Li-Dong Wang

**Affiliations:** 1 Henan Key Laboratory for Esophageal Cancer Research of the First Affiliated Hospital, Zhengzhou University, Zhengzhou, Henan, China; 2 Cancer Research Center and the Third Affiliated Hospital, Xinxiang Medical University, Xinxiang, Henan, China; 3 Department of Thoracic Surgery, Anyang Tumor Hospital, Anyang, Henan, China; 4 Department of Pathology, the Second Affiliated Hospital of Zhengzhou University, Zhengzhou, Henan, China; Kagoshima University Graduate School of Medical and Dental Sciences, Japan

## Abstract

**Background:**

The human major histocompatibility complex (MHC) is the most important region in vertebrate genome, and is crucial in innate immunity. Recent studies have demonstrated the possible role of polymorphisms in the MHC region to high risk for esophageal squamous cell carcinoma (ESCC). Our previous genome-wide association study (GWAS) has indicated that the MHC region may confer important risk loci for ESCC, but without further fine mapping. The aim of this study is to further identify the risk loci in the MHC region for ESCC in Chinese population.

**Methods:**

Conditional logistic regression analysis (CLRA) was performed on 24 single nucleotide polymorphisms (SNPs) within the MHC region, which were obtained from the genetically matched 937 cases and 692 controls of Chinese Han population. The identified promising SNPs were further correlated with clinical and clinicopathology characteristics. Immunohistochemistry was performed to explore the protein expression pattern of the related genes in ESCC and neighboring normal tissues.

**Results:**

Of the 24 promising SNPs analyzed, we identified three independent SNPs in the MHC region associated with ESCC: rs35399661 (P = 6.07E-06, OR = 1.71, 95%CI = 1.36–2.17), rs3763338 (P = 1.62E-05, OR = 0.63, 95%CI = 0.50–0.78) and rs2844695 (P = 7.60E-05, OR = 0.74, 95%CI = 0.64–0.86). These three SNPs were located at the genes of HLA-DQA1, TRIM27, and DPCR1, respectively. Further analyses showed that rs2844695 was preferentially associated with younger ESCC cases (P = 0.009). The positive immunostaining rates both for HLA-DQA1 and TRIM27 were much higher in ESCC tissues than in neighboring normal tissues (69.4% vs. 26.8% for HLA-DQA1 and 77.6% vs. 47.8% for TRIM27, P<0.001). Furthermore, the overexpression of HLA-DQA1 is correlated significantly with age (P = 0.001) and family history (P<0.001).

**Conclusion:**

This study for the first time provides evidence that multiple genetic factors within the MHC region confer risk to ESCC on the subjects from high-risk area in northern China.

## Introduction

Esophageal cancer (EC) is one of the most aggressive gastrointestinal malignancies and prevalent in the developing world [1]. The rural regions around the Taihang Mountain in the junction of Henan, Hebei, and Shanxi provinces in northern China have been well documented as the highest incidence areas for EC [2]-[4] _ENREF_1_ENREF_1. There are two main histological types for EC, squamous cell carcinoma (ESCC) and adenocarcinoma, each with distinct etiological and pathological characteristics [5]_ENREF_4. ESCC is the predominant histological type worldwide,especially in northern China, comprising more than 90% of all EC cases [6]_ENREF_5. The pathogenesis of ESCC is complex, involving both environmental and genetic risk factors. Recent genome-wide association studies (GWAS) have identified couple of susceptibility loci for ESCC in Han Chinese [7]–[9]. Assessing the individual genetic susceptibility can help identify high risk patients with a potential better benefit from the surveillance programs [10]. Our previous GWAS [9] has indicated that major histocompatibility complex (MHC) regions may confer important risk loci for ESCC, but without further fine mapping.

The MHC, human leukocyte antigen (HLA) system, is the most important region in vertebrate genome with respect to autoimmunity, and is crucial in innate immunity [11] _ENREF_9. MHC genes are located in a gene-dense region of the human genome on Chromosome 6p21.31 and the complex is organized from centromere to telomere as classes I, II, and III. Lack of HLA presentation has been proposed to contribute to the immune evasion of cancer cells in some cancers including EC [12]_ENREF_10. Although several studies have demonstrated that the genetic variants in MHC region is associated with an increased risk to ESCC [12]–[14], the association of GWAS with MHC in ESCC has not been well characterized.

The present study, thus, was undertaken to fine mapping the association signal within MHC region with susceptibility to ESCC with conditional logistic regression analysis (CLRA) on our previous GWAS of ESCC in Chinese population [9]. The identified promising SNPs were further correlated with gender, age, family history, alcohol consumption, smoking status and clinicopathology characteristics. Immunohistochemistry was performed to explore the protein expression pattern of the related genes in ESCC and neighboring normal tissues.

## Materials and Methods

### Ethics Statement

The study was approved by the ethical review committee of Zhengzhou University and conducted according to Declaration of Helsinki principles. Written informed consent was obtained from all the participants.

### Recruitment of patients and controls

All the 1,089 patients with ESCC and the 1,763 normal control subjects were from the high incidence area in northern China and obtained from Endoscopic Screening Centers within multiple hospitals for early detection of ESCC. The enrollment criteria for the cases and controls in this study were the same as described in our previous GWAS study [9]_ENREF_11_ENREF_11. In brief, there were no restrictions on age, gender, ethnicity, risk factor exposures or cancer-stages for cases or controls; local residents in the same geographic areas; all the cases were histopathologically confirmed ESCC and no prior history of treatment for ESCC other than surgery; the tumor location, gross type, degree of differentiation, the regional lymph node metastasis and clinicopathological stage were recorded based on the International Statistical Classification of Diseases and Related Health Problems (10th Revision, WHO, 2006, online); all the controls subjects in this study, without any family history of ESCC, were undergone esophageal endoscopic examination to exclude the early ESCC.

### SNP genotyping

Genomic DNAs were extracted from peripheral blood leukocytes using a standard procedure using Flexi Gene DNA kits (QIAGEN, Germany) as previously described [9]. Briefly, DNA concentration was normalized to 50 ng/μl (diluted in 10 mM Tris/L mM EDTA) with a Nanodrop Spectrophotometer (ND-1000). Approximately 200 ng of genomic DNA was used for genotyping analysis. Each sample was whole-genome amplified, fragmented, precipitated and resuspended in appropriate hybridization buffer. Denatured samples were hybridized on prepared Illumina Human 610-Quad BeadChips. After hybridization, the BeadChips oligonucleotides were extended by a single labeled base, which was detected by fluorescence imaging with an Illumina Bead Array Reader. Normalized bead intensity data obtained for each sample were loaded into the Illumina BeadStudio 3. The genome-wide genotyping analysis was conducted using Illumina Human 610-Quad BeadChips at the Key Laboratory of Dermatology (Anhui Medical University), Ministry of Education, China, Hefei, Anhui, China. All genotyping passed through our quality control procedure and there was no significant deviation from Hardy-Weinberg proportions in control population.

As previous described [9], the GWAS was carried out in 1,089 ESCC cases and 1,763 controls of the subjects in northern China using Illumina Human 610-Quad BeadChips. After SNPs and samples based quality control filtering, 506,666 SNPs as well as 1,077 cases and 1,733 controls were left for further analyses.

### Principal components analysis

As described in our previous GWAS [9], principal components analysis (PCA) was used twice in our data. In brief, firstly, PCA of our 2,810 GWAS samples (1,077 cases and 1,733 controls) alone or in combination with 206 reference HapMap samples was performed. The HapMap samples were drawn from: Yoruba in Ibadan, Nigeria (YRI) (n = 57), Japanese in Tokyo, Japan (JPT) (n = 44), Han Chinese in Beijing, China (CHB) (n = 45) and CEPH (Utah residents with ancestry from northern and western Europe) (CEU) (n = 60). This analysis identified 1,181 genetically unmatched subjects. After removal these samples, we got 1629 matched subset samples. Second, 1,629 GWAS samples (937 cases and 692 controls) alone or in combination with 206 reference HapMap samples was performed. The genome-wide χ2 inflation factor λ was reduced from 1.505 to 1.075 after removal of the 1181 unmatched samples, suggesting that population structure was not a major confounder in the matched-sample analysis.

### Selection criteria of SNPs

All the 6,252 SNPs in MHC region (Chr. 6: 25–34 Mb) were obtained for further study based on the 1,629 samples (937 cases and 692 controls). Among these SNPs, 1049 SNPs failed in missingness test >0.1; 2,607 SNPs failed in frequency test (MAF<0.05), and 163 SNPs failed in Hardy-Weinberg equilibrium (HWE) test (p<0.001). Finally, 3,515 SNPs in 937 ESCC cases and 692 controls were left for downstream analysis. Simultaneously, we selected the first-choice set of promising SNPs for further analysis based on the considerations of each SNP in terms of the P value, frequency of minor allele and biological characteristics of the related genes in carcinogenesis. Finally, 24 SNPs from 17 loci were selected for downstream analysis.

### Clinicopathological characteristics measurement

Smoking status was defined as never, former and current smokers. Alcohol consumption was defined as nondrinkers who reported never drinking and drinkers who drank regularly in the 1 year prior to age at diagnosis for cases. Subjects were asked to report whether any first-, second-, and third-degree relatives had been diagnosed with other upper gastrointestinal tract cancers, including EC, gastric cancer or gastric cardia adenocarcinoma. Positive family history of EC was defined as occurrence at least 2 upper gastrointestinal tract cancer cases within three degree relatives, which was the same as we previously reported [9]. The tumor location was recorded based on the International Statistical Classification of Diseases and Related Health Problems (10th Revision, WHO, 2006, online) and was classified as upper (20 to 25 cm from incisors), middle (>25 to 30 cm from incisors) and lower thoracic (>30 to 40 cm from incisors) (7th Edition of the AJCC).

### Immunohistochemical staining in ESCC and neighboring normal tissues

To understand the protein expression pattern in the tissues, serial paraffin block tissue sections were obtained from of the surgically resected ESCC specimen and neighboring normal tissues. Sixty-seven ESCC cases with 60 matched normal tissues (7 pieces of tissue were off-chip during the histological dehydration process) for TRIM27 immunostaining and 49 ESCC cases with 41 matched normal tissues (8 pieces of tissue were off-chip during the histological dehydration process) for HLA-DQA1 immunostaining (ProteinTech Group, Inc., Chicago, USA). All the patients were from Linzhou, Henan Province, the high incidence area for EC in northern China and had not received treatment other than surgery. The avidin-biotin-peroxidase complex (ABC) method was applied as previously described [15]_ENREF_12. In brief, after dewaxing, inactivating, endogenous peroxidase activity and blocking cross-reactivity with pre-immune serum, the sections were incubated over night at 4°C with the primary antibodies (antibody for TRIM27 was diluted at 1∶150 and the one for HLA-DQA1 was 1∶100). Localization of the primary antibodies was achieved by subsequent incubation of a biotinylated anti-primary antibody, an avidin-biotin complex conjugated to horseradish peroxidase, and diaminobenzidine (Vectastain Elite Kit). The slides were washed 3 times with phosphate-buffered saline (PBS) after incubation. As negative controls, some slides were subjected to normal serum blocking and omission of the primary antibody. Intense nuclear or cytoplasm staining was the criterion for a “positive” reaction. We applied the criteria established by our laboratory previously [15] to describe the patterns of positive result as follows: “scattered”, in which only some isolated positive cells were identified; “papillary”, where immunostain-positive cells were identified only in the esophageal epithelial papillary area; “focal”, where wide clusters of positive cells were seen in some areas of the epithelia; and “diffuse”, in which the sheets of positive cells were found throughout most areas of the lesions. Two co-authors, the experienced pathologists (L-D W and J-L R) were specified to read the immunostaining slides.

### Statistical analysis

The conditional logistic analysis (CLRA) with backwards selection was applied in this study to identify the strongest associated SNP by the SAS statistical software package (SAS Institute Inc., Cary, NC). All the 24 SNPs identified from the GWAS study were taken as independent parameters in this CLRA model. ORs and 95% CIs were calculated as estimates of the relative risk for variables. Data were analyzed with PLINK 1.06, STATISTICAL ANALYSIS SYSTEM (SAS) version 8.1 and SPSS 17.0 (SPSS Inc, Chicago, IL). The Bonferroni corrected P value based on multiple testing of 3,515 SNPs and was set at 0.05. In all stratification analysis, comparisons of allele frequencies were made using the Pearson's χ^2^ test and the statistical significance was set at P<0.05.

## Results

### SNP genotyping and quality control and CLRA

**Figure 1:**
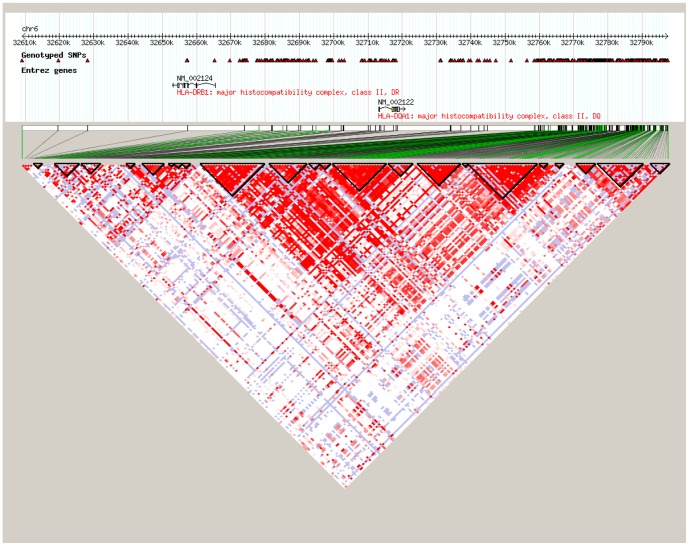
The LD pattern of signal I at 6p21.31 in Asian population (CHB+JPT).

**Figure 2:**
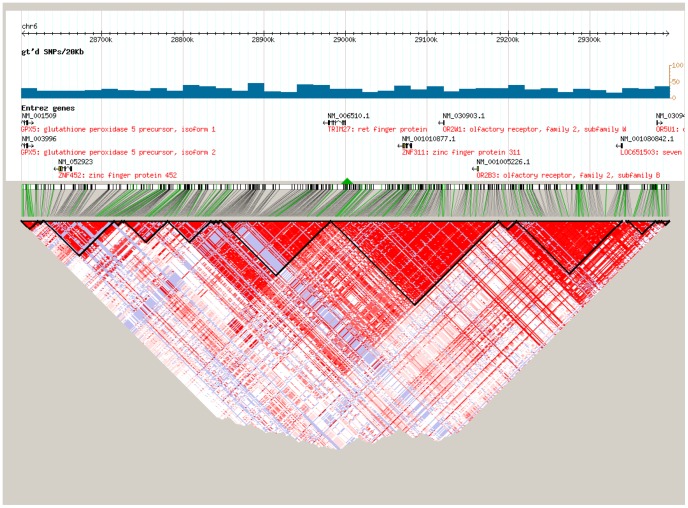
The LD pattern of signal II at 6p21.31 in Asian population (CHB+JPT).

**Figure 3:**
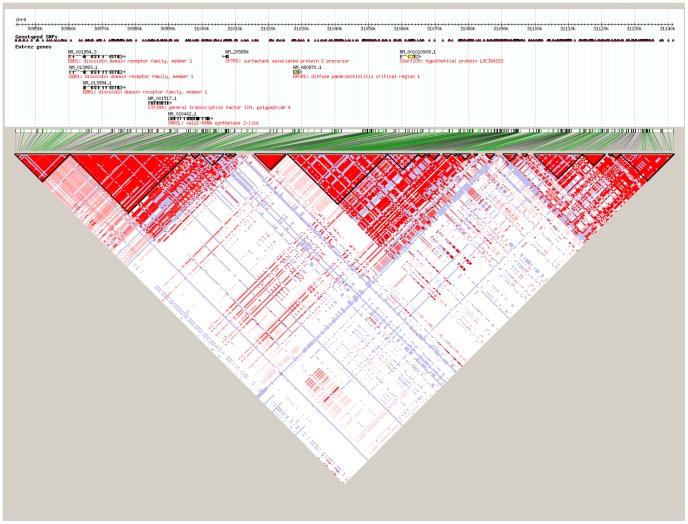
The LD pattern of signal III at 6p21.31 in Asian population (CHB+JPT).

After strict quality control to the SNPs, the 24 promising SNPs selected for association testing are listed in Table 1 along with the Bonferroni corrected P values based on multiple testing of 3,515 SNPs. Since SNP rs17533090 was highly correlated with SNP rs35399661 and could be completely represented by the latter (D’ = 1/r^2^ = 1 in HapMap CHB data), only the rs35399661 locus was kept for further statistical analysis. Of the 24 promising SNPs analyzed, we identified three independent SNPs in the MHC region that associated with ESCC through CLRA: rs35399661 (P = 6.07E-06, OR = 1.71, 95%CI = 1.35–2.17), rs3763338 (P = 1.62E-05, OR = 0.63, 95%CI = 0.50–0.78) and rs2844695 (P = 7.60E-05, OR = 0.74, 95%CI = 0.64–0.86) (Table 1). The Bonferroni corrected P values for rs35399661, rs3763338 and rs2844695 were 0.021, 0.057 and 0.267, respectively (Table 1). These three SNPs in fact represent three independent loci containing multiple candidate genes which lie within each linkage disequilibrium (LD) block on chromosome 6p21.31 (Figures 1-3), which were located at the genes of HLA-DQA1, TRIM27, and DPCR1 (Table 1). Signal I surrounding rs35399661 covers two (HLA-DQA1 and HLA-DRB1) genes (Figure 1); Signal II surrounding rs3763338 covers four (TRIM27, ZNF311, OR2W1 and OR2B3) genes (Figure 2); Signal III surrounding rs2844695 covers two (DPCR1 and C6 or f205) genes (Figure 3).

**Table 1:** Summary of association results and conditional logistic regression analysis of the 24 SNPs.

							MAF^b^					CLRAf		CLRA^g^		CLRA^h^	
Gene	SNP	BP	Loction*	Distance#	LD&	Alleea	F_Ac	F_Ud	P value	P^e^ value	OR (95%CI)	OR	P value	OR	P value	OR	P value
HLA-G	rs35540400	29822923	F*-3UTR	24024	0	T	0.46	0.40	1.73E-04	0.608	1.32 (1.14-1.52)	1.35	9.71E-05	1.35	1.01E-04	1.30	1.17E-03
MICC	rs1264524	30404786	F-5UTR	17243	0	A	0.43	0.50	5.67E-05	0.199	0.74 (0.64-0.86)	0.75	1.94E-04	0.76	2.74E-04	0.77	1.17E-03
ITPR3	rs3818528	33661091	intron	/	0	G	0.11	0.08	7.44E-04	1	1.52 (1.19-1.95)	1.49	2.14E-03	1.49	2.24E-03	1.53	2.04E-03
HLA-DPB2	rs9277723	33091454	intron	/	0	A	0.39	0.46	7.54E-05	0.265	0.74 (0.64-0.86)	0.73	6.00E-05	0.74	1.36E-04	0.79	3.49E-03
IP6K3	rs9469583	33717770	F-5UTR	3044	0	A	0.14	0.09	1.95E-04	0.685	1.53 (1.22-1.91)	1.53	2.78E-04	1.51	4.44E-04	1.43	3.99E-03
DDR1	rs2517576	30815035	F-5UTR	35659	0.038	A	0.13	0.09	7.15E-04	1	1.47 (1.18-1.84)	1.49	6.50E-04	1.44	1.67E-03	1.45	4.52E-03
DDR1	rs2844659	30824532	F-5UTR	26162	0.042	A	0.13	0.09	7.15E-04	1	1.47 (1.18-1.84)	1.49	6.50E-04	1.44	1.67E-03	1.45	4.52E-03
DDR1	rs2844657	30829522	F-5UTR	21172	0.042	G	0.13	0.09	8.04E-04	1	1.47 (1.17-1.84)	1.48	7.39E-04	1.44	1.86E-03	1.45	5.06E-03
MLN	rs2281820	33768897	coding	/	0	A	0.19	0.14	1.39E-04	0.489	1.45 (1.20-1.76)	1.43	3.24E-04	1.42	4.87E-04	1.35	5.14E-03
HMGN4	rs9379888	26526994	F-5UTR	11578	0	A	0.13	0.08	2.95E-05	0.104	1.67 (1.31-2.13)	1.70	2.34E-05	1.69	3.10E-05	1.42	6.64E-03
DPCR1	rs11753326	30925985	F-3UTR	3987	0.034	G	0.13	0.09	6.25E-04	1	1.49 (1.18-1.87)	1.48	9.54E-04	1.43	2.85E-03	1.39	1.11E-02
HLA-DQA1	rs9271300	32581582	F-5UTR	23587	0.015	C	0.45	0.51	2.26E-04	0.794	0.77 (0.67-0.88)	0.84	2.76E-02	0.84	2.17E-02	0.84	3.46E-02
NOTCH4	rs482759	32195017	F-5UTR	3173	0.039	G	0.11	0.08	6.74E-04	1	1.52 (1.19-1.94)	1.35	2.24E-02	1.31	3.88E-02	1.26	9.73E-02
NOTCH4	rs483574	32194956	F-5UTR	3112	0.039	A	0.11	0.08	6.74E-04	1	1.52 (1.19-1.94)	1.35	2.24E-02	1.31	3.88E-02	1.26	9.73E-02
C6orf100	rs9257403	28916972	F-3UTR	4657	0.064	G	0.47	0.41	4.35E-04	1	1.29 (1.12-1.48)	1.30	4.69E-04	1.20	1.81E-02	1.12	1.73E-01
OR12D3	rs9380120	29335537	F-3UTR	5663	0.782	G	0.08	0.12	1.49E-04	0.524	0.64 (0.51-0.81)	0.65	3.38E-04	0.86	3.73E-01	0.82	2.46E-01
C6orf100	rs2071788	28912307	3UTR	/	0.827	A	0.08	0.12	4.75E-04	1	0.67 (0.53-0.84)	0.66	5.16E-04	1.44	2.49E-01	1.41	3.16E-01
DDX6P1	rs9357078	29297666	3UTR	/	0.765	A	0.08	0.12	2.10E-04	0.738	0.65 (0.51-0.82)	0.65	4.30E-04	0.88	4.39E-01	0.84	3.16E-01
OR2B3	rs4713201	29067263	F-5UTR	12173	0.529	G	0.09	0.13	7.64E-04	1	0.68 (0.54-0.85)	0.68	1.45E-03	1.05	8.12E-01	1.02	9.29E-01
OR2J3	rs9295794	29104935	F-3UTR	24274	0.012	A	0.09	0.13	8.70E-04	1	0.68 (0.55-0.86)	0.69	1.70E-03	1.04	8.23E-01	0.99	9.70E-01
DPCR1	rs2844695	30936014	F-3UTR	14016	/	G	0.38	0.45	7.60E-05	0.267	0.74 (0.64-0.86)	0.72	2.27E-05	0.72	2.43E-05	NA	NA
TRIM27	rs3763338	28894311	F-5UTR	2543	/	A	0.10	0.14	1.62E-05	0.057	0.63 (0.50-0.78)	0.62	2.15E-05	NA	NA	NA	NA
HLA-DQA1	rs35399661	32590990	F-5UTR	14179	/	G	0.13	0.08	6.07E-06	0.021	1.71 (1.36-2.17)	NA	NA	NA	NA	NA	NA
HLA-DQA1	rs17533090	32590722	F-5UTR	14447	/	A	0.13	0.08	7.74E-06	0.027	1.70 (1.34-2.14)	NA	NA	NA	NA	NA	NA

*Flanking;

# the distance of the SNP to gene;

& the LD (r^2^) of each SNP with the top SNP in HapMap;

a effect allele;

b Minor allele frequency;

c Frequency of minor allele in cases;

d Frequency of minor allele in controls;

e Bonferroni corrected P value based on multiple testing of 3,515 SNPs;

Conditional regression analysis to (^f^) rs35399661, (^g^) rs35399661and rs3763338, and (^h^) rs35399661, rs3763338 and rs2844695.

### The effects of SNPs on clinical characteristics

To further correlate the three promising SNPs identified above with clinicopathological characteristics of ESCC, we performed stratified analyses of rs35399661, rs3763338 and rs2844695 by gender, age, family history, alcohol consumption, smoking status, tumor location, gross type, cancer cell differentiation, regional lymph node metastasis and pathological staging (Tables 2–3). The χ^2^ test of each subgroup showed the rs2844695 was preferentially associated with younger ESCC cases (P = 0.009, OR = 1.32, 95%CI = 1.07–1.62) (Table 2). However, the other clinicopathological characteristics did not show significant association with these three promising SNPs (Tables 2–3), indicating the potential values of these SNPs in clinic.

**Table 2:** Stratified analyses of rs35399661, rs3763338 and rs2844695 by the clinical characteristics in ESCC.

	rs35399661				rs3763338				rs2844695			
Clinical characteristics (n)	F_A^b^	?2	P	OR (95%CI)	F_A^c^	?2	P	OR (95%CI)	F_A^d^	?2	P	OR (95%CI)
Sex												
male (550)	0.12	2.970	0.085	0.79 (0.60–1.03)	0.09	0.109	0.082	0.95 (0.70–1.03)	0.38	0.001	0.978	0.68 (0.52–0.90)
female (387)	0.15				0.10				0.38			
Age												
>50 (847)	0.13	0.863	0.353	0.82 (0.53–1.25)	0.10	3.681	0.055	1.89 (0.98–3.64)	0.40	7.207	0.007	1.32 (1.07–1.62)
≤50 (90)	0.13				0.05				0.34			
Family history												
positive (284)	0.13	0.012	0.914	0.98 (0.74–1.32)	0.10	0.128	0.971	1.06 (0.76–1.48)	0.36	1.867	0.172	0.87 (0.71–1.06)
negative (645)	0.13				0.09				0.39			
Drinking												
drinkers (106)	0.11	1.295	0.255	0.77 (0.49–1.21)	0.11	0.367	0.262	1.16 (0.72–1.86)	0.42	1.673	0.196	1.22 (0.90–1.64)
nondrinkers (620)	0.14				0.10				0.38			
Smoking												
smokers (178)	0.13	0.148	0.701	0.93 (0.66–1.33)	0.11	0.499	0.719	1.15 (0.78–1.71)	0.41	1.571	0.210	1.17 (0.92–1.50)
nonsmokers (548)	0.14				0.09				0.38			

a Minor allele (A) frequency of rs17533090 in cases;

b Minor allele (G) frequency of rs35399661 in cases;

c Minor allele (A) frequency of rs3763338 in cases;

d Minor allele (G) frequency of rs2844695 in cases; HIA: high-incidence area; LIA: low-incidence area. df = 1.

**Table 3:** Stratified analyses of rs35399661, rs3763338 and rs2844695 by the clinicopathology characteristics in ESCC.

	rs35399661			rs3763338			rs2844695		
Clinicopathology characteristics (n)	F-A^b^	?2	P	F-A^c^	?^2^	P	F-A^d^	?2	P
Tumor location#									
upper thoracic (67)	0.17	3.592	0.166	0.14	3.166	0.205	0.42	0.274	0.254
middle thoracic (228)	0.11			0.09			0.39		
lower thoracic (53)	0.14			0.08			0.32		
Gross type*									
MT (149)	0.11	1.018	0.759	0.07	4.812	0.17	0.39	0.198	0.978
UT (121)	0.14			0.12			0.38		
ST (10)	0.10			0.15			0.35		
FT (31)	0.13			0.06			0.39		
DD#									
PD (122)	0.12	2.909	0.228	0.12	0.635	0.723	0.39	1.471	0.479
MD (229)	0.13			0.10			0.37		
WD (17)	0.03			0.09			0.28		
Regional lymph node^&^									
positive (104)	0.13	0.111	0.739	0.09	0.377	0.539	0.36	0.419	0.518
negative (226)	0.14			0.10			0.39		
Clinicopathological stage#									
early (39)	0.17	0.761	0.683	0.08	0.996	0.608	0.47	4.387	0.112
moderate (195)	0.14			0.11			0.37		
late (80)	0.13			0.09			0.34		

a Minor allele (A) frequency of rs17533090 in cases;

b Minor allele (G) frequency of rs35399661 in cases;

c Minor allele (A) frequency of rs3763338 in cases;

d Minor allele (G) frequency of rs2844695 in cases;

MT: medullary type; UT: ulcerative type, ST: scirrhous type;

FT: fungating type;

DD: degree of differentiation;

PD: poorly-differentiated;

MD: moderate-differentiated;

WD: well-differentiated.

# df = 2; *df = 3; ^&^df = 1

### Immunohistochemical analysis for TRIM27 and HLA-DQA1 protein

**Figure 4:**
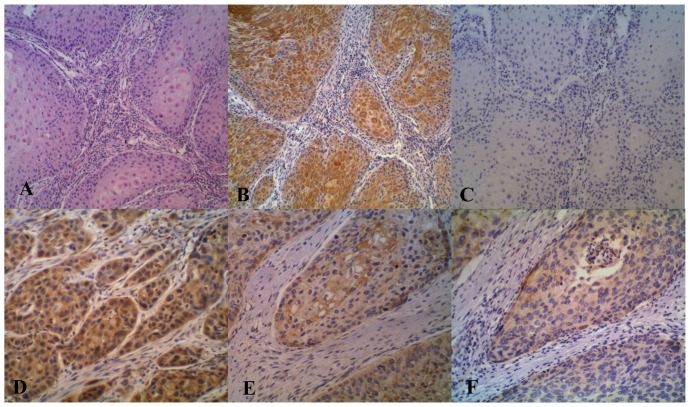
The hematoxylin-eosin staining (A, 10X) and immunohistochemical staining for TRIM27 (B, 10X and E, 20X) and HLA-DQA1 (D, 20X and F, 20X) on ESCC tissue. C (10X) was the negative control of A (omission of the first antibody application). A, B and C were the serial tissue sections from the same ESCC patient. E and F were also from the same patient.

To further demonstrated the clinical relevance of the two SNPs showing the association with ESCC, expression of TRIM27 (for rs3763338) and HLA-DQA1 (for rs35399661) proteins in ESCC and the neighboring normal tissues was determined using immunohistochemical analysis (Figure 4). Marked expression of TRIM27 was observed in 77.6% of ESCC sections as compared with 47.8% of neighboring normal tissues (P<0.001). HLA-DQA1 staining was seen in 69.4% of ESCC samples versus 26.8% of neighboring normal tissues (P<0.001). Expression of TRIM27 and HLA-DQA1 was analyzed with the stratification of different sex, age, family history, high/low-incidence areas, the regional lymph node metastasis and tumor location (Tables 4 and 5). The overexpression of HLA-DQA1 was correlated significantly with age (P = 0.001) and family history (P<0.001) (Table 5). There was no correlation between TRIM27 expression and clinical characteristics (Table 4).

**Table 4:** Relationship of TRIM27 protein immunostaining and clinical characteristics of ESCC.

Clinical characteristics	N	Positive staining n (%)	?^2^	P	OR (95%CI)
Gender					
Male	33	28 (84.8)	1.960	0.162	0.43 (0.13–1.43)
Female	34	24 (70.6)			
Age#					
≤60	28	22 (78.6)	0.025	0.873	0.91 (0.28–2.93)
>60	39	30 (76.9)			
Family history#					
positive	13	9 (69.2)	0.652	0.419	1.74 (0.45–6.71)
negative	54	43 (79.6)			
Area* #					
HIA	52	40 (76.9)	0.063	0.801	1.20 (0.29–5.00)
LIA	15	12 (80)			
Regional lymph node#					
positive	16	10 (62.5)	2.763	0.96	2.80 (0.81–9.70)
negative	51	42 (82.4)			
Tumor location^&^					
upper thoracic	13	11 (84.6)	2.54	0.281	/
middle thoracic	44	36 (81.8)			
lower thoracic	4	2 (50)			

*HIA: high-incidence area; LIA: low-incidence area.

# df = 1; ^&^df = 2

**Table 5:** Relationship of HLA-DQA1 protein immunostaining and clinical characteristics of ESCC.

Clinical characteristics	N	Positive staining n (%)	?^2^	P	OR (95%CI)
Gender#					
Male	22	14 (63.6)	0.622	0.430	0.61 (0.18–2.08)
Female	27	20 (74.1)			
Age#					
≤60	25	12 (48)	10.992	0.001	0.08 (0.02–0.44)
>60	24	22 (91.7)			
Family history#					
positive	39	7 (17.9)	20.856	0.000	0.10 (0.03–0.28)
negative	39	27 (69.2)			
Area* #					
HIA	37	27 (73.0)	0.914	0.339	0.52 (0.13–2.02)
LIA	12	7 (58.3)			
Regional lymph node#					
positive	13	7 (53.8)	1.467	0.226	1.86 (0.48–7.23)
negative	36	26 (72.2)			
Tumor location^&^					
upper thoracic	9	8 (88.9)	1.453	0.528	/
middle thoracic	31	22 (71.0)			
lower thoracic	3	2 (66.7)			

* HIA: high-incidence area; LIA: low-incidence area.

# df = 1; ^&^df = 2.

## Discussion

Three GWAS of ESCC conducted among Chinese Han populations have been published [7]–[9]. Though previous studies have investigated the role of individual genetic variation in the etiology of esophageal cancers, the effect of high density SNP genotypes of the MHC region in the etiology of ESCC is still unclear, and little genome-wide data are available. Strengths of this study include that it is conducted in high-incidence areas with highly homogeneous population and that the controls are well-matched with ESCC cases with similar environmental exposures.

In the present study, we have performed the first GWAS of ESCC in the MHC region on the subjects from high risk area in northern China and found three important independent susceptibility loci containing three biologically interesting candidate genes, i.e., HLA-DQA1, TRIM27 and DPCR1. TRIM27, also known as ret finger protein (RFP), which is characterized by a conserved RING finger, a B-box, and a coiled-coil domain (together called RBCC) [17], [18], encodes a member of the tripartite motif (TRIM) family [19]_ENREF_18. Currently, the biological function of RFP has not been characterized; however, many of the TRIM family members sharing the RBCC moiety participate in the control of cell survival [19]_ENREF_18. It has been shown that the RBCC moiety is required for the transforming capacities of these TRIM oncogenes_ENREF_19. The role of RFP influences apoptotic pathways [19]_ENREF_18 and acquire oncogenic activity when fused to kinases by chromosomal rearrangements [20]_ENREF_20. Compared to many normal tissues, cancer cells are highly sensitized to apoptotic signals, and survive only because they have acquired lesions [21]_ENREF_21. Indeed, immunohistochemical analysis for TRIM27 in our study demonstrated the higher positive rate of the TRIM27 protein in ESCC samples than in neighboring normal epithelia (P<0.001). Therefore, RFP is likely to play an important role in esophageal carcinogenesis through modulating apoptotic pathways.

HLA-DQA1 belongs to the HLA class II alpha chain paralogues. The class II molecule is a heterodimer consisting of an alpha (DQA) and a beta chain (DQB), both anchored in the membrane. HLA-DQA1 plays a central role in the immune system by presenting peptides derived from extracellular proteins [22]_ENREF_22. Class II molecules are expressed in antigen presenting cells (B lymphocytes, dendritic cells and macrophages). Within the DQ molecule both the alpha chain and the beta chain contain the polymorphisms specifying the peptide binding specificities, resulting in up to four different molecules. MHC class II molecules bind peptides derived from proteins that have entered the endocytic pathway and presented them at the cell surface for interaction with CD4^+^ T cells [23]_ENREF_23. The presence of HLA-DQ expression in premalignant lesions and on some tumor cells appears to confer an advantage to the host in terms of restricted tumor growth [24]_ENREF_24 and survival [24] _ENREF_24through their role as initiators of CD4+ T helper cell responses against the tumor. Simultaneously, our preliminary immunohistochemical analysis showed that the positive rate of HLA-DQA1 staining in ESCC samples was apparently higher than the neighboring normal epithelia. The results further supported the potential role of HLA-DQA1 in esophageal carcinogenesis.

DPCR1, also known as diffuse panbronchiolitis critical region 1, located between HLA-B and HLA-A on chromosome 6p21.33, is classified as one of the MHC class I molecules. The DPCR1 gene may contain markers for diagnosis of diffuse pan-bronchiolitis, a bronchiolar disease that affects human airways [25]. However, to our knowledge, the association between DPCR1 variations and risk of ESCC has not yet been investigated. Our findings may be meaningful for future studies validating the function of DPCR1. Interestingly, the LD pattern of Signal III (Figure 3) showed that rs2844695 was an independent locus.

Previous studies have indicated that smoking, alcohol consumption, aging and gender are risk factors for ESCC [26], [27]_ENREF_27_ENREF_25. Our results demonstrated that rs2844695 was preferentially associated with younger ESCC cases. The other factors (gender, age, family history, alcohol consumption, smoking, tumor location, gross type, degree of differentiation, the regional lymph node metastasis and pathological stage) did not significantly alter the effects ofrs35399661 and rs3763338 on the risk to ESCC, indicating that the three SNPs identified as most promising from our study could provide orthologous information to existing clinicophthological covariates.

We also examined the correlation of TRIM27 and HLA-DQA1 expression in ESCC with clinical characteristics. Our results showed that the expression of TRIM27 and HLA-DQA1 was higher in ESCC tissues than in neighboring normal tissues. The results further supporting the potential role of TRIM27 and HLA-DQA1 in esophageal carcinogenesis. The expression of HLA-DQA1 was correlated with age and family history of ESCC with the older ESCC cases having higher HLA-DQA1 expression than the younger ones. Furthermore, the percentage of patients expressing HLA-DQA1 is higher for negative family history than positive family history. The result suggested that HLA-DQA1 may be potential in inhibiting the malignant properties in ESCC.

The limitation of the present study is the absence of replication in another independent samples for rs35399661 (HLA-DQA1 genes), rs3763338 (TRIM27 genes) and rs2844695 (DPCR1 genes). Interestingly, recent studies have demonstrated that polymorphisms occurred in HLA-DQA1 increase the risk and prognosis to lung squamous cell carcinoma [28] and gastric cancer [29]. Further replication studies would be desirable to elucidate the role of these SNPs on the susceptibility, and possibly in the prognosis for ESCC.

### Conclusion

This study for the first time provides evidence that multiple genetic factors within the MHC region confer risk to ESCC on the subjects from high-risk area in northern China. Further dissection of the roles of these loci will likely to lead to insights into the etiology of this rapidly evolving and fatal cancer.
